# Pentacam corneal topography and densitometry features of PCOS patients

**DOI:** 10.1186/s12886-022-02758-4

**Published:** 2023-01-03

**Authors:** Tugce Gizem Cengiz Ozturk, Hanife Tuba Akcam, Aski Ellibes Kaya

**Affiliations:** 1 Department of Ophthalmology, Duzce University School of Medicine, Duzce, Turkey; 2 Department of Ophthalmology, Duzce State Hospital, Duzce, Turkey; 3 Department of Ophthalmology, Ankara Yıldırım Beyazıt University School of Medicine, Ankara, Turkey; 4 Department of Obstetrics and Gynecology, Duzce University School of Medicine, Duzce, Turkey; 5 Department of Obstetrics and Gynecology, Private Clinic, Samsun, Turkey

**Keywords:** Cornea, Corneal densitometry, Corneal ectasia, Corneal topography, Hormones, Inflammation, Ovary, Pentacam, Polycystic ovary syndrome

## Abstract

**Background:**

To evaluate corneal topography and densitometry features in patients with polycystic ovary syndrome (PCOS) and compare them with healthy individuals.

**Methods:**

53 eyes of 53 female patients diagnosed with PCOS and 53 eyes of 53 age-matched female volunteers were analyzed in the study. In addition to the detailed ophthalmological and gynecological examination, anterior segment analysis was performed using Pentacam. A complete analysis of aberrometric, keratometric, topometric, and, densitometric values between the groups was performed, and the results were outlined.

**Results:**

According to the results, although Kmax-front, Kmean-front, ISV, IVA, IHA, BAD_D and PI-Avg values were slightly higher in PCOS group along with a slight thinning in the thinnest location, there was no statistically significant difference between the groups. Moreover, correlation analysis between PCOS clinical parameters and keratometric/topometric/aberrometric data were found to be almost normal. Yet, when Pentacam tomography maps of all cases are examined in detail, mild ectatic changes were observed in 5 cases in PCOS group. Furthermore, a significant increase in thickness across all densitometry values except anterior (10–12 mm), central (10–12 mm), and total (10–12 mm) was found in PCOS group.

**Conclusions:**

Our study showed that an intensification of corneal densitometry values ​​and various changes in keratometry data implying ectasia can be observed in patients with PCOS. Prospective studies with larger patient series are needed to reveal any potential relationship between PCOS and corneal abnormalities.

## Introduction

Polycystic ovary syndrome (PCOS) is a chronic multisystemic disease which afflicts females of reproductive age. It is also the most common gynecological endocrinopathy with its worldwide prevalence of approximately 5–15%. Although the root-cause and working mechanism of PCOS, whose onset is typically in adolescence, is not fully disclosed, it was found that many pathogenetic mechanisms such as genetic predisposition and environmental factors, especially molecular mechanisms and the inflammation pathway, take part in determining the clinical course of the syndrome [1]. In addition to the well-known systemic features of PCOS, it’s local effects on many eye structures such as the changes on the ocular surface were reported recently [2, 3]. Ocular surface is a multifunctional unit consisting of lid margin, tear film, cornea, and conjunctiva. Of these components, cornea is very sensitive to environmental factors, and any changes on the cornea are of vital importance in terms of the potential to affect optical quality [4].

Recent corneal imaging techniques provide more information on abnormalities in corneal tissue enabling early diagnosis and treatment [5]. Among those methods, corneal Scheimpflug tomography helps in detecting both the changes of the curve and height on the surface of anterior and posterior cornea as well as offering multipoint pachymetry and densitometry information. Combined topographic, pachymetric and densitometric information is more valuable as it allows early diagnosis of keratoconus (KC) with high sensitivity and specificity [6].

Our aim in this study is to address a hiatus in the literature by exploring the Pentacam corneal topography and densitometry characteristics of patients with PCOS and comparing them with healthy individuals.

## Methods

Duzce University's Ethical Committee (IRB#2019–256) reviewed and approved the study, and HIPAA criteria and the tenets of the Declaration of Helsinki were also conformed. Participants were informed about the nature and implications of the study and their consent was obtained during the first clinical visit.

### Study population

Our study enrolled 53 eyes of 53 female patients with PCOS (PCOS group) and 53 eyes of 53 age-matched healthy female volunteers (Control group) resulting in a confidence level of 95% and error margin of 4.12%.

Patients recently diagnosed with PCOS in the obstetrics and gynecology clinic of Duzce University hospital were included in PCOS group while female patients who applied to the Duzce University hospital ophthalmology outpatient clinic for routine control or refraction examination and were known to be ocular/systemically healthy volunteers were assigned to control group upon receiving their consent.

Exclusion criteria are consisted by the following: presence of any systemic (i.e. autoimmune, metabolic, hormonal, inflammatory, neurological) diseases (except polycystic ovary syndrome in PCOS group), pregnancy, any acute/chronic eye diseases (i.e. keratoconus, dry eye diseases, corneal dystrophies, cataract, glaucoma, uveitis) except low grade refractive error, history of previous eye surgery, histories of systemic/ocular trauma/injury, contact lens usage, long-term use of topical drugs, spherical refractive error more than plus/minus 4 diopter (in terms of spherical equivalent refraction), and astigmatism more than plus/minus 0.75 diopter. Only Turkish patients were included in the study to eliminate any potential effects of race on the study results. Besides, only the right eyes were analyzed and no consanguinity between the participants were allowed.

### Eye examination

Refractive error measurement (Topcon RM-8800 Autorefractometer; Topcon Corporation, Tokyo, Japan), best corrected visual acuity (Snellen) assessment, corrected intraocular pressure determination by pneumatic tonometer (Canon TX-20 P; Canon Inc, Tokyo, Jap), slit-lamp examination, and fundus evaluation (SL-120; Carl Zeiss, Germany) were performed for all participants. Moreover, anterior segment imaging was carried out using Pentacam Scheimpflug (Oculus Optikgeräte GmbH Inc., Wetzlar, Germany) where keratometric, topometric, aberrometric and densitometric measurements were obtained in the 8 mm zone in a single session.

### Pentacam Scheimpflug topography and densitometry measurement

The same (experienced and oblivious to subject’s PCOS status) physician performed the imaging with the Scheimpflug camera (Pentacam, OCULUS Optikgeräte, Germany) by (T.G.C.O) during the same hours of day (1:00–3:00 p.m.) under standard dim-light conditions and without dilating the pupil.

Following the vendor’s recommended settings, Pentacam measurements were obtained by using Pentacam software version 1.21r41. Patients’ chin and forehead were suitably positioned and then they were made blink before keeping both eyes open and gaze fixated to the target. After ensuring adequate alignment, 25 images of each eye were captured and 12,500 elevation points were recorded by the camera in 2 s. To eliminate any measurement errors due to poor imaging, 3 repetitions per eye were made, and the best image in terms of alignment and fixation was included for statistical analysis. Only high-quality images evaluated by the device confirmation (“OK” statements) were considered valid for the study.

In evaluating corneal aberrations, wavefront measurements were taken from the 6 mm zone so that the pupil size did not affect the readings. Total root mean square (RMS) and RMS high-order aberration (HOA) values were recorded via Zernike analysis.

Corneal densitometry measurements (12-mm corneal diameter) were completed using densitometry feature of Pentacam yielding densitometric values at three different depths consisting anterior (120-μm thick- the superficial region of the cornea), central (located between anterior and posterior layer), and posterior layer (60-μm thick—the innermost region of the cornea). Besides, the corneal area was further divided into four concentric zones as first, second, third, and fourth depending on their respective 0–2 mm, 2–6 mm, 6–10 mm and 10–12 mm diameters of the annular area. Depending on the degree of backscattering light from the cornea, the measurements ranged from 0 (maximum transparency) to 100 (minimum transparency to total corneal opacity).

### PCOS examination and classification

PCOS patients were diagnosed by an experienced obstetrician and gynecologist (A.E.K) applying Revised Rotterdam criteria (2004) [7]. The diagnosis was followed by PCOS classification based on the clinical and laboratory characteristics of the patients. In line with the single blinded study principles, the practicing obstetrician could not see the results of eye measurements until the patient was recruited for the study.

### Statistical analysis

SPSS 22.0 (SPSS Inc, Chicago, IL, USA) software was used for statistical analysis and normality of the data was tested by the Shapiro–Wilk procedure. Normally distributed values were presented as the mean ± standard deviation (SD) where non-normally distributed values were presented as the median (IQR, interquartile range: Q3-Q1). Since the data that will be subjected to pairwise comparison were found to be normally distributed, independent samples t-test was suitable for the analysis.

Additionally, Spearman correlation coefficients were used to test for correlation. Statistical significance level was set at P < 0.05 and the results were evaluated within the 95% confidence interval for the entire study.

## Results

The groups were similar in terms of age as the means for PCOS group and control group were 26.40 ± 6.94 and 25.79 ± 6.67 years respectively (p = 0.649). While the spherical equivalent refraction rates were -0.98 ± 1.30 and -0.86 ± 1.11 diopter in PCOS and control groups (p = 0.602); corrected intraocular pressure values were 14.02 ± 2.92 mmHg and 13.74 ± 2.45 respectively (p = 0.591). Clinical phenotypes of the PCOS group showed that 30 of the patients were obvious, 7 of them were classic, 2 of them were ovulatory and, 14 of them were mild PCOS. Median DHEA-SO4 value was 2.32 μg/ml (IQR = 0.175), and median total testosterone value was 0.82 ng/ml (IQR = 0.33) in the PCOS group.

Besides, according to keratometric data, although PCOS group had slightly higher Kmax-front (44.50 ± 1.52 D versus 44.21 ± 1.40 D) and Kmean-front (43.37 ± 1.38 D versus 43.23 ± 1.29 D) compared to the control group; thinnest location value was thinner (539 ± 33.50 µm versus 541.92 ± 27.44 µm) yet with no statistical and clinical significance. Moreover, despite lacking statistical significance, topometric indicators such as surface variability index (ISV), vertical asymmetry index (IVA), height asymmetry index (IHA), Belin/Ambrosio enhanced ectasia total deviation value (BAD_D) and mean progression index (PI-Avg) were slightly higher in the PCOS group. Remaining topometric data also followed the suit. In addition to that, unlike aberrometric indexes which were similar between the groups (Table 1), all parameters of corneal densitometry data were statistically significantly higher in PCOS group except anterior, central, and total densitometry values in 10–12 mm (Table 2).

**Table 1:** Keratometric, topometric and aberrometric indices

Parameters	PCOS Group	Control Group	*P* value^a^
Kmax (front) (D)	44.50 ± 1.52	44.21 ± 1.40	0.319
Thinnest location (µm)	539 ± 33.50	541.92 ± 27.44	0.624
Kmean (front) (D)	43.37 ± 1.38	43.23 ± 1.29	0.597
Kmean (back) (D)	-6.31 ± 0.22	-6.32 ± 0.23	0.801
ISV	16.94 ± 4.11	15.53 ± 4.66	0.100
IVA (mm)	0.13 ± 0.05	0.12 ± 0.05	0.357
KI	1.01 ± 0.02	1.01 ± 0.01	0.866
CKI	1.0 ± 0.006	1.0 ± 0.005	0.090
IHA (µm)	5.26 ± 3.84	4.72 ± 3.78	0.472
IHD (µm)	0.01 ± 0.005	0.01 ± 0.006	0.755
Rmin	7.56 ± 0.32	7.64 ± 0.25	0.156
BAD_D	1.05 ± 0.71	0.90 ± 0.58	0.246
PI-Avg	1.06 ± 0.15	1.05 ± 0.12	0.815
RMS total (front)	1.73 ± 0.51	1.72 ± 0.48	0.962
RMS HOA (front)	0.43 ± 0.10	0.43 ± 0.10	0.763
RMS total (back)	0.81 ± 0.15	0.76 ± 0.15	0.168
RMS HOA (back)	0.21 ± 0.04	0.20 ± 0.04	0.538

Abbreviations: *BAD_D *Belin/Ambrósio enhanced ectasia total deviation value, *CKI *Central keratoconus index, *HOA *Higher-order aberration, *IHA *Index of height asymmetry, *IHD I*ndex of height decentration, *ISV *Index of surface variance, *IVA *Index of vertical asymmetry, *KI *Keratoconus index, *PI-Avg *Progression index average, *Rmin *Minimum radius of curvature, *RMS *Root mean square

^a^Independent samples t-test. Data were presented as mean ± standard deviation

**Table 2:** Corneal densitometry values

**Zones**		**PCOS Group**	**Control Group**	***P*** value
0-2 mm	Anterior	19.15 ± 2.08	17.01 ± 2.35	0.0001*
	Center	14.66 ± 1.56	13.26 ± 1.43	0.0001*
	Posterior	11.93 ± 1.33	10.90 ± 1.0	0.0001*
	Total	15.26 ± 1.59	13.73 ± 1.56	0.0001*
2–6 mm	Anterior	17.51 ± 1.96	15.55 ± 1.98	0.0001*
	Center	13.28 ± 1.41	12.03 ± 1.15	0.0001*
	Posterior	11.03 ± 1.27	10.09 ± 0.88	0.0001*
	Total	13.94 ± 1.49	12.55 ± 1.31	0.0001*
6–10 mm	Anterior	17.28 ± 3.30	14.85 ± 2.32	0.0001*
	Center	13.83 ± 2.35	12.07 ± 1.60	0.0001*
	Posterior	11.90 ± 1.82	10.48 ± 1.18	0.0001*
	Total	14.41 ± 2.46	12.47 ± 1.66	0.0001*
10–12 mm	Anterior	21.63 ± 5.69	20.94 ± 6.03	0.546
	Center	18.98 ± 3.71	17.61 ± 4.08	0.07
	Posterior	15.86 ± 2.89	14.38 ± 3.14	0.01*
	Total	18.82 ± 3.75	17.65 ± 4.12	0.128
Total	Anterior	18.35 ± 2.53	16.36 ± 2.23	0.0001*
	Center	14.60 ± 1.82	13.10 ± 1.49	0.0001*
	Posterior	12.23 ± 1.51	11.02 ± 1.19	0.0001*
	Total	15.06 ± 1.87	13.49 ± 1.58	0.0001*

^*^, *P* < 0.05. Abbreviations: mm, milimeters

^a^Independent samples t-test. Data were presented as mean ± standard deviation

Furthermore, Kmean (back) value was positively correlated with the level of total testosterone (P = 0.016); however, there was no significant correlation between aberrometric indices and clinical data of PCOS patients (Table 3); and between corneal densitometry values and clinical data of PCOS patients in the same fashion (Table 4).

**Table 3:** Correlation between demographic data and keratometric/topometric/aberrometric indices in the PCOS group

Parameters	Clinical Phenotype of PCOS		DHEA-SO4		Total Testosterone	
	Rho	P^a^	Rho	P^a^	Rho	P^a^
Kmax (front)(D)	-0.007	0.963	-0.083	0.557	0.236	0.088
Thinnest location (µm)	-0.042	0.768	-0.150	0.283	-0.058	0.681
Kmean (front)(D)	-0.095	0.499	-0.087	0.536	0.255	0.065
Kmean (back)(D)	-0.061	0.665	-0.067	0.633	0.329	0.016*
ISV	0.080	0.571	-0.079	0.573	0.062	0.658
IVA (mm)	0.003	0.983	-0.085	0.545	-0.034	0.811
KI	0.068	0.628	-0.067	0.635	0.066	0.639
CKI	-0.094	0.503	-0.187	0.179	0.225	0.105
IHA (µm)	0.081	0.563	0.041	0.768	-0.012	0.934
IHD (µm)	0.088	0.531	0.092	0.512	-0.015	0.913
Rmin	-0.059	0.677	0.137	0.327	-0.213	0.125
BAD_D	0.003	0.986	-0.044	0.755	0.215	0.122
PI-avg	0.025	0.856	0.015	0.913	0.227	0.102
RMS total (front)	0.257	0.063	0.104	0.457	-0.035	0.804
RMS HOA (front)	0.195	0.163	-0.120	0.393	-0.90	0.522
RMS total (back)	-0.048	0.735	-0.148	0.292	0.182	0.191
RMS HOA (back)	0.045	0.749	-0.156	0.266	0.178	0.202

Abbreviations: *Rho* Spearman's rank correlation coefficient

^a^Spearman’s correlation test. *, *P* < 0.05

**Table 4:** Correlation between demographic data and corneal densitometry values in the PCOS group

**Zones**		**Clinical Phenotype of PCOS**		**DHEA-SO4**		**Total Testosterone**	
		Rho	P^a^	Rho	P^a^	Rho	P^a^
0–2 mm							
	Anterior	-0.125	0.373	-0.105	0.454	0.038	0.789
	Center	-0.108	0.442	-0.028	0.840	0.148	0.292
	Posterior	-0.102	0.466	-0.058	0.680	0.146	0.297
	Total	-0.101	0.472	-0.079	0.574	0.104	0.460
2–6 mm							
	Anterior	-0.059	0.676	-0.136	0.332	0.008	0.952
	Center	-0.033	0.816	-0.096	0.494	0.115	0.413
	Posterior	-0.029	0.838	-0.138	0.325	0.115	0.411
	Total	-0.044	0.752	-0.121	0.389	0.080	0.570
6–10 mm							
	Anterior	0.051	0.716	-0.196	0.160	-0.015	0.914
	Center	0.083	0.557	-0.211	0.130	0.057	0.685
	Posterior	0.064	0.649	-0.248	0.074	0.152	0.278
	Total	0.101	0.470	-0.214	0.124	0.057	0.683
10–12 mm							
	Anterior	0.108	0.439	-0.126	0.368	-0.089	0.526
	Center	0.096	0.495	-0.121	0.388	0.054	0.703
	Posterior	0.002	0.986	-0.133	0.343	0.136	0.333
	Total	0.083	0.552	-0.150	0.283	-0.011	0.935
Total							
	Anterior	0.010	0.941	-0.170	0.225	-0.063	0.654
	Center	0.076	0.586	-0.183	0.189	0.110	0.434
	Posterior	0.028	0.843	-0.228	0.101	0.200	0.150
	Total	0.052	0.712	-0.176	0.208	0.048	0.735

Abbreviations: *Rho* Spearman’s rank correlation coefficient

^a^ Spearman’s correlation test. *, *P* < 0.05

**Fig. 1:**
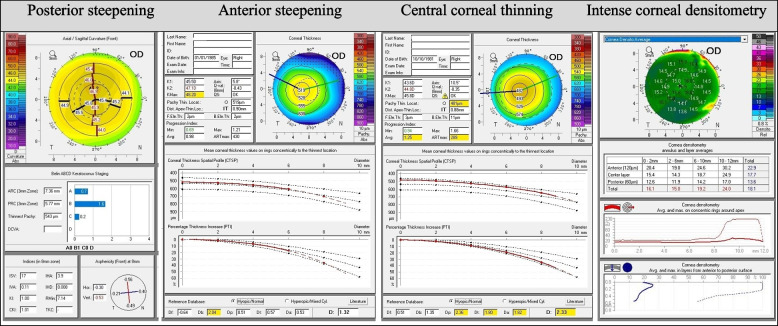
Abnormal Pentacam views of PCOS patients

Last but not least, a comprehensive examination of Pentacam individual tomography maps of all participants in the light of clinical features exhibited some mild pathological Pentacam changes which may be associated with ectasia in 5 cases in the PCOS group. That’s to say that, there was an isolated posterior steepening in 1 eye (PRC = 5.77 mm), an isolated anterior steepening in 1 eye (anterior Kmax = 48.2), and an isolated anterior central corneal thinning in 3 eyes (pachymetry values ​​at the thinnest point = 482, 489 and 494 µm) (Fig. 1).

## Discussion

The cornea, an important element of the ocular surface, is a transparent layer with optical properties [8].Vision can be severely affected by acquired and/or congenital disorders of this layer. While some of these diseases show early symptoms, some of them are hard to detect already advancing to final stages when diagnosed. Of these, ectatic diseases of the cornea, especially KC, have particular importance in terms of screening [9] because, in addition to the standard complete ophthalmological examination, corneal topography test which is a non-invasive and reliable imaging technique mapping the corneal surface [10] is often needed to diagnose them.

Although the true etiology of KC is not explored yet, a number of pathogenic factors such as atopy, genetic predisposition, environmental factors, oxidative stress, hormonal status are believed to play role and its association with some systemic conditions such as connective tissue diseases, Ehlers Danlos syndrome and pregnancy [11, 12] is well-established. Contrary to its former categorization as a non-inflammatory disease, recent studies showed that KC may be associated with inflammation. For example, inflammatory mediators such as reactive oxygen products, IL-6, and TNF-alpha were found to be increased in patients with KC [13]. Likewise, the heightened risk of KC in such diseases as rheumatoid arthritis, systemic lupus erythematosus, Hashimoto's thyroiditis, ulcerative colitis and psoriasis with chronic inflammation adds to evidence that the disease may have inflammatory origins [14, 15].

PCOS is a multisystemic disease with inflammatory and autoimmune origins [16]. Since the main factor that initiates the physiological changes in PCOS is the excess of androgen hormone, it can be said that it is a hormonal disease. Number of studies showed that the cornea is also affected by such periods as menstrual cycle, pregnancy, and menopause as they cause hormonal fluctuations and thus affect hormone receptors on the cornea [17–19] When it comes to ocular effects in PCOS, ocular surface disorders are the prominent ones. Some recent studies in the literature draw attention to the close relationship between PCOS, dry eye and meibomian gland dysfunction [20, 21].

Moreover, changing values of keratometric/topometric parameters in diseases with steroid hormone excess are also studied. For instance, Dutta et al. [22] detected KC in a patient with adrenal myelolipoma with chronic adrenal stimulation and attributed it to the chronically high steroid hormone levels. McKay et al.[23], on the other hand, found high levels of dehydroepiandrosterone sulfate (DHEA-S) in the salivary secretions of KC patients regardless of gender. In fact, they emphasized that the increase in DHEA-S levels boosts the release of cytokines in the immune system and cytokine hypersecretion in turn plays a role in the pathogenesis of KC, and they suggested that DHEA-S levels can also be used as a biomarker for early diagnosis of KC or in risk assessment of KC.

As a matter of fact, since the anatomical regularity of the collagen fibrils, the integrity of the connective tissue and the balanced keratocyte elements are important factors for clarity of the cornea, it can be assumed that some systemic diseases may alter the corneal density measurement even in the absence of corneal clouding or damage [24]. As corneal transparency decreases, densitometry values ​​increase. Nowadays, corneal densitometry gained importance in the follow-up/treatment of many ocular and systemic diseases. For example, it was showed that corneal densitometry values ​​increase in many systemic diseases including gout, lichen planus, multiple myeloma and psoriasis [25].

Considering the above information, it is possible that the surface properties of the cornea, and therefore keratometric, topometric, aberrometric and densitometric values, are affected in patients with PCOS whose etiopathogenesis includes both inflammatory and hormonal disorders. Nevertheless, there is not a study in the literature yet evaluating Pentacam corneal tomography test data in patients with PCOS making our study the first of its kind.

Further to above results, even though no direct relationship was found between PCOS and KC in our study, there was a statistically significant difference in corneal densitometry values and a potential inclination towards ectasia was discovered. Nonetheless, sampling from newly diagnosed PCOS patients, low average age and relatively small study size may have limited more far fetching insights from our topography analysis. However, the fact that corneal densitometry results were significant despite being in the early stages of the disease is a point deserving special attention.

Omission of corneal hysteresis evaluation can be considered as another shortcoming of our study. Although the evaluation could not be made because the clinic did not have an ocular response analyzer device, we believe that such a study would be very useful in order to better analyze the biomechanical changes in the cornea.

## Conclusions

Our study shed light on the various changes in Pentacam tomographic maps of PCOS patients. We believe that the results obtained through our study will ignite interest in the scientific community, lead prospective studies on larger patient series and disclose potential cause-and-effect mechanisms.

## Data Availability

The data that support the findings of this study are available from the corresponding author upon reasonable request.
